# Supplementary material for: MetaRibo-Seq measures translation in microbiomes
Source: Nat Commun. 2020 Jun 29;11:3268. doi: 10.1038/s41467-020-17081-z (PMC7324362; doi:10.1038/s41467-020-17081-z)
Supplement: Supplementary file 10 — Supplementary Data 7 [file 41467_2020_17081_MOESM10_ESM.zip › File2/Confidence_High_Taxonomy/93747_out.krona.html]

Javascript must be enabled to view this page.

members
magnitude
magnitudeUnassigned
count
unassigned
taxon
rank

93747\_out

2

2
superkingdom
2

2
phylum
1239

1
91061
class

1
order
186826

1
family
1300

genus
1301
1


SRS013723\_contig\_number\_44810
1
1305
species

1
class
186801

1
order
186802

1
541000
family

1
genus
1263


SRS046717\_contig\_number\_contig-100\_3862.3863
1
657323
species
